# Supplementary material for: The effect of recall period on reported out-of-pocket health expenditure in Ghana
Source: PLoS One. 2025 Dec 19;20(12):e0290910. doi: 10.1371/journal.pone.0290910 (PMC12716721; doi:10.1371/journal.pone.0290910)
Supplement: S1 Fig — (DOCX) [file pone.0290910.s001.docx]

**Fig 1: Design of Household Health Survey Instrument**

Health Provider records

Validation
