# Supplementary material for: The effect of recall period on reported out-of-pocket health expenditure in Ghana
Source: PLoS One. 2025 Dec 19;20(12):e0290910. doi: 10.1371/journal.pone.0290910 (PMC12716721; doi:10.1371/journal.pone.0290910)
Supplement: S2 Table — (DOCX) [file pone.0290910.s002.docx]

**Supplementary material 2**

# **Table S1: Spending categories by recall periods**

| **Level of *D*isaggregation - 11 Health items** | **Recall periods:**  **D- days/ M-Months** | | | | |
| --- | --- | --- | --- | --- | --- |
|  | **15d** | **30d** | **3M** | **6M** | **12M** |
| **COICOP code 06.2.3 Inpatient care services** |  |  |  |  |  |
| **1.1 Medical treatment and / or care that required overnight stay in a nursing home; ( medical convalescent homes; palliative care establishments) or any other long term care medical facility** |  |  |  | **X** | **X** |
| **1.2. Medical and dental treatment that required an overnight stay in any type of facility** (e.g. hospitals, clinics) **excluding long term care medical facility** |  |  |  | **X** | **X** |
| Includes payments for all medical services, diagnostic and laboratory tests , medicines and medical products needed during the overnight stay. Also include emergency transportation services and emergency rescue.  Excludes: non-emergency transportation and non-medical costs for patient’s relative. |  |  |  |  |  |
| **COICOP code 06.2.1 Preventive care services** |  |  |  |  |  |
| **2.1. Immunization/vaccination services including for maternal and child care** |  |  | **X** | **X** |  |
| Includes ; travel and tourism vaccination as well as any other immunization/vaccination service. |  |  |  |  |  |
| **2.2. Other preventive services such as prenatal/postnatal care, child growth and development visits, family planning, screening, tests, consultations to detect communicable or non-communicable diseases before symptoms appear** (e.g. diabetes, heart problems) |  |  | **X** | **X** |  |
| Includes diagnostic and laboratory tests needed to provide preventive services but exclude payments for the vaccine itself when separately invoiced from the service. |  |  |  |  |  |
| **COICOP code 06.4.2 Emergency transportation and emergency rescue** |  |  |  |  |  |
| **3.1 Patient emergency transportation services and emergency rescue services (excluding those associated with an overnight stay)** | **X** | **X** |  |  |  |
| Excludes non-emergency transportation services |  |  |  |  |  |
| **COICOP code 06.2.2 & 06.2.3 Outpatient dental & other outpatient services** |  |  |  |  |  |
| **3.2 Dental consultations that did not require an overnight stay; including dental diagnostics services and laboratory tests needed to provide outpatient dental services** (e.g. X-rays, blood tests) | **X** | **X** |  |  |  |
| For any illness, disease, injury or health problem; from any type of provider; inside or outside a hospital setting |  |  |  |  |  |
| **3.3 other medical consultations ; diagnostics services and laboratory tests that did not require an overnight stay** | **X** | **X** |  |  |  |
| Includes any diagnostic and laboratory test needed to provide outpatient medical services (e.g. X-rays, blood/urine tests), but excludes emergency transportation services and emergency rescue |  |  |  |  |  |
| **COICOP code 06.1.1** |  |  |  |  |  |
| **4.1 Herbal medicines (tablets or syrups) and homeopathic products for consumption outside a health facility or institution.** | **X** | **X** |  |  |  |
| **4.2 Medicines (branded, generic ), vaccines, oral contraceptives, vitamins and minerals for consumption outside a health facility or institution.** | **X** | **X** |  |  |  |
| **COICOP code 06.1. 2 medical diagnostic products, prevention and protective devices** |  |  |  |  |  |
| **5.** (Pregnancy tests, incontinence products and absorbent including diapers for the aging population, inhalers, mechanical contraceptives; insecticide treated mosquito nets, blood pressure devices) **and other medical health products** **for personal use** **outside a health facility or institution**. | **X** | **X** |  |  |  |
| Includes repair, rental and maintenance |  |  |  |  |  |
| **COICOP code 06.1.3 Assistive products for vision, hearing, mobility and daily living.** |  |  |  |  |  |
| **6. Purchase, repair, rental/maintenance of** (glasses for vision; hearing aids; crutches & wheelchairs; therapeutic footwear; walkers; pressure relief mattresses ) **and all other assistive health products** . | **X** | **X** |  |  |  |

# **Table S2: structure and wording of health expenditure questions in survey questionnaire**

| **Questions** | **Instructions for the CAPI version** |
| --- | --- |
| **Now I am going to ask some questions about medical payments. Include all formal and informal payments for all services and products for any member of your household.** | - All words in bold are compulsory. - Do not change the focus of this section which is about payments for health care. |
| **Q1: Over the past …. did you or any member of your household make any payment for …..?**  **1.1 Medical treatment and / or care that required overnight stay in a nursing home;** ( medical convalescent homes; palliative care establishments) **or any other long term care medical facility? (6/12 months)**  **1.2 medical or dental treatment that required an overnight stay**  **in any type of facility excluding long term care medical facility (6/12 months )**  **2.1 Immunization/vaccination services including for maternal and child care (3/6 months)**  **2.2 other preventive services such as prenatal/postnatal care,** child growth and development visits, **family planning, screening, tests, medical consultations to detect** communicable or non-communicable **diseases before symptoms appear (e.g. diabetes, heart problems)? (3/6 months)**  **3.1 patient emergency transportation services and emergency rescue (excluding those associated with an overnight stay) (15/30 days)**  **3.2 Dental services that did not require an overnight stay (15/30 days)**  **3.3**  **other medical consultations and services than dental and preventive that did not require an overnight stay (15/30 days)**  **4.1 Herbal medicines (tablets or syrups) for consumption outside a health facility or institution 15/30 days**  **4.2 Medicines** (branded, generic), **vaccines, oral contraceptives, vitamins and minerals for consumption outside a health facility or institution (15/30 days)**  **5. Pregnancy tests,** blood pressure devices, inhalers, mechanical contraceptives; incontinence products, absorbent including diapers for the aging population; insecticide treated mosquito nets **and all other medical/protective/treatment device for personal use outside a health facility or institution. 15/30 days**  **6. Purchase, repair, rental/maintenance of glasses for vision,** contact lenses**; hearing aids; Crutches & wheelchairs;** therapeutic footwear; walkers; pressure relief mattresses **and all other assistive health products** for vision, hearing mobility and daily living **for use outside a health facility/institution. (6/12 months)** | - Ask all questions for each of the products and services - **Questions Q1 to Q5 should be asked for all items.** - **Start with first item and ask questions Q1 to Q5. Continue then with the next item etc…** |
| 1.Yes  2. No |  |

| **Questions** | **Instructions for the CAPI version** |
| --- | --- |
| **Q2: If yes what was the total amount paid?**  **1.1 Please include payments for all medical services, diagnostic and laboratory tests , medicines and medical products, emergency transportation and emergency rescue services needed during the overnight stay in a long term care facility**  **1.2 Please include payments for all medical services, diagnostic and laboratory tests , medicines and medical products, emergency transportation and emergency rescue services needed during the overnight stay**  **2.1 Please exclude payments for the vaccine itself when separately invoiced from the service.**  **2.2. Please include diagnostic and laboratory tests needed to provide preventive services.**  **3.1 Please exclude non-emergency transportation**  **3.2 Please include diagnostic and laboratory tests needed to provide dental services**  **3.3 Please include diagnostic and laboratory tests needed to provide other medical services than dental and preventive**  **5.**  **Includes repair, rental and maintenance**  Enter value (amount) | Specify the inclusion/exclusion criteria where relevant. E.g. for 1.2  If yes what was the total amount paid? Please include payments for all medical services, diagnostic and laboratory tests , medicines and medical products, emergency transportation and emergency rescue services needed during the overnight stay |
| **Q3a:**  **Which of the following sources did you use to pay for such amount?**  **1. household’s income**  **2. remittance or money gift**  **3. cash savings**  **4. selling of any household’s assets or goods (housing, land, animals, jewellery, appliances or machines)**  **5. barter of household assets or goods**  **6. loan**  **7. insurance/government program**  **9. Other source of funding that those I have just asked you about (specify)**     1. Yes   Enter source if 9 >>> Q3b   1. No >> next source of funding unless code 9, move to Q4 | - Ask Q3a for each source of funding listed under 1 to 9 one by one. Start with code 1 and move to Q3b if there is a positive answer. If the answer is negative then ask about code 2. - For source 9: specify the source. So you need to program this option. - For source 1, if the answer is yes don’t ask the amount - After source 9 if answer is “no” move to Q4 |
| **Q3b: If yes (in Q3a) What was the total amount financed from…**  **2. remittance or money gift**  **3. cash savings**  **4. selling of any household’s assets or goods (housing, land, animals, jewelry, appliances or machines)**  **5. barter of household assets or goods**  **6. loan >>>3c**  **7. insurance/government program**  **9. selling harvest in advance or relied on any other source of funding that those I have just asked you about (specify)**  Enter value (amount)  DK/NA…88888 | - If answer is 6. Loan, ask 3c - Any other answer go to next source listed in 3a - After source 9. Move to Q4 |
| **Q3c: What was the type of loan?**  1 no interest rate, no collateral  2 with both interest rate and collateral  3 with either interest rate or collateral  DK/NA…88888 | - Ask if one of the sources is 6. Loan |
| **Q4. Have you or any member of your household received …. without making any payment over the past….?**  1.Yes >> Q5  2. No >> next item |  |
| **Q5. Over the past…., if you had to pay for ….received how much would that have cost you?**  Enter value (amount) |  |
