# Supplementary material for: The effect of recall period on reported out-of-pocket health expenditure in Ghana
Source: PLoS One. 2025 Dec 19;20(12):e0290910. doi: 10.1371/journal.pone.0290910 (PMC12716721; doi:10.1371/journal.pone.0290910)
Supplement: S4 Fig — (DOCX) [file pone.0290910.s004.docx]

**Fig 2: Sample size and matching summary**

**Version-1** (2wks/6months)

11 Health items, 42 non-health items

**Version-2** (4wks/12months)

11 Health items, 42 non-health items

Total households interviewed=746 household sample=670

Provider sample =76

% OOPs = 386 (48)

% OOPs matched = 283(73)

**Inpatient care**

Household report=108

Number matched to provider=35 (32%)

**Preventive care**

Household report=28

Number matched to provider=20 (71%)

**Other health services**

Household report=0

Number matched to provider=0 (0%)

**Outpatient care**

Household report=33

Number matched to provider=20 (61%)

**Medicines**

Household report=295

Number matched to provider=234 (79%)

**Medical products**

Household report=9

Number matched to provider=0 (0%)

Total households interviewed =480

Household sample=431

Provider sample=49

% OOPs = 279 (58)

% OOPs matched = 226(81)

**Inpatient care**

Household report=100

Number matched to provider=64 (64%)

**Preventive care**

Household report=21

Number matched to provider=15 (71%)

**Other health services**

Household report=1

Number matched to provider=0 (0%)

**Outpatient care**

Household report=15

Number matched to provider=11 (73%)

**Medicines**

Household report=204

Number matched to provider=167 (82%)

**Medical products**

Household report=2

Number matched to provider=1 (50%)
