# Supplementary material for: The effect of recall period on reported out-of-pocket health expenditure in Ghana
Source: PLoS One. 2025 Dec 19;20(12):e0290910. doi: 10.1371/journal.pone.0290910 (PMC12716721; doi:10.1371/journal.pone.0290910)
Supplement: S5 Table — (DOCX) [file pone.0290910.s005.docx]

**Supplementary Results**

**Table 1: Households with out-of-pocket payments by spending category for matched household on reported expenditures**

|  | **Questionnaire Version 1** | | | | | **Questionnaire Version 2** | | | | |
| --- | --- | --- | --- | --- | --- | --- | --- | --- | --- | --- |
|  | **(Short recall period)** | | | | | **(Long recall period)** | | | | |
| **Spending category** | Recall period | Households with positive OOPs  N=722 | | **Number and proportion matched** | | Recall period | Households with positive OOPs  N=431 | | **Number and proportion matched** | |
|  |  | n | % | n | % |  | n | % | n | % |
| inpatient care services | 6 months | 89 | 12 | 17 | 19 | 12 months | 53 | 12 | 19 | 36 |
| preventive services | 3 months | 18 | 3 | 12 | 67 | 6 months | 19 | 5 | 13 | 68 |
| Other health  services | 2 weeks | 0 | 0 | 0 | 0 | 4 weeks | 1 | 0.23 | 0 | 0 |
| Outpatient | 2 weeks | 25 | 4 | 17 | 68 | 4 weeks | 10 | 2 | 8 | 80 |
| Medicines | 2 weeks | 278 | 39 | 222 | 80 | 4 weeks | 185 | 43 | 147 | 79 |
| health products | 6 months | 5 | 0.7 | 0 | 0 | 12 months | 1 | 0.23 | 1 | 100 |

**Table 2 Comparison of mean OOPs in household samples by spending category**

|  | **Questionnaire**  **Version 1** | | | **Questionnaire**  **Version 2** | | |  |
| --- | --- | --- | --- | --- | --- | --- | --- |
|  | **(short recall period)** | | | **(Long recall period)** | | | Ratio (mean v1/mean v2) (95% CI) |
|  | n | mean | sd | N | mean | sd |  |
| **Inpatient** | 89 | 478 | 1726 | 53 | 404 | 806 | 1.18 (0, 2.45) |
| **Medicines** | 278 | 15 | 44 | 185 | 18 | 38 | 0.82 (0.42, 1.23) |
| **Outpatient** | 25 | 46 | 83 | 10 | 24 | 21 | 2.21 (0, 5.18) |
| **Preventive care** | 18 | 24 | 31 | 19 | 98 | 253 | 0.19 (0, 0.66) |
| **Other medical services** | 0 | 0 | 0 | 1 | 200 | - | - |
| **Health products** | 5 | 21 | 21 | 1 | 4 | - | 5.3 (0.60, 10) |

**Table 3: Mean bias and variability in measurement of OOPs by recall period**

| Spending category | Number of observations | Mean bias | 95% limits of agreement | Estimated difference in bias (qu2 vs qu1) & CI & p-value | Estimated difference in SD (qu2 vs qu1 ( & CI & p-value |
| --- | --- | --- | --- | --- | --- |
| **Medicines** | | | | | |
| 2 weeks recall period (qu1) | 222 | 1.38 | 0.40 – 4.77 | - | - |
| 4 weeks recall period (qu2) | 147 | 1.35 | 0.37 – 4.92 | 1.04 (0.82 – 1.33) 0.73 | 1.02 (0.86 – 1.21) 0.84 |
