# Supplementary material for: The effect of recall period on reported out-of-pocket health expenditure in Ghana
Source: PLoS One. 2025 Dec 19;20(12):e0290910. doi: 10.1371/journal.pone.0290910 (PMC12716721; doi:10.1371/journal.pone.0290910)
Supplement: Attachment — Submitted filename: Feedback OOP.docx [file pone.0290910.s007.docx]

Feedback for Authors

Generally, the article seeks a worthy course but some comments must be addressed as shown below;

The effect of a recall period on reported out-of-pocket health expenditure in Ghana

Abstract

Authors should state the implications of their findings.

Background

The background of the study requires a very strong justification. Why was the study done in the study area? This should come out strongly in the background. The last paragraph of the background statement should be moved to the study setting.

Methods

The figure under randomization should be given a title and the source of it well provided. Reference should be made to the figure before introducing it.

Providers

Please, are you talking about health facilities or health providers?

Healthcare financing in the study setting

Issues about healthcare financing should be moved to the study setting.

Out-of-pocket payments in the study setting

Issues about out-of-pocket payments should either be moved to the study setting or the background information.

Results

Authors should check the labelling of the tables due to inconsistencies identified during the review.

Conclusions

Please, state how relevant the findings are or what are the implications of the study findings.

There are typographical issues that must be addressed. Therefore, proofreading the article will be very helpful.

Best wishes
