# Supplementary material for: The effect of recall period on reported out-of-pocket health expenditure in Ghana
Source: PLoS One. 2025 Dec 19;20(12):e0290910. doi: 10.1371/journal.pone.0290910 (PMC12716721; doi:10.1371/journal.pone.0290910)
Supplement: Attachment — Submitted filename: Response to Reviewers.docx [file pone.0290910.s009.docx]

**Response to editor and reviewers’ comments**

Below is a detailed response to the comments raised by the editor and reviewers. For clarity, the comments have been disaggregated according to the points raised by the reviewers and addressed accordingly.

**Abstract**

**Comment**: Authors should state the implications of their findings.

**Response:** The abstract has now been updated to reflect the implications of the findings

**Background**

**Comment:** The background of the study requires a very strong justification. Why was the study done in the study area? This should come out strongly in the background**.** The last paragraph of the background statement should be moved to the study setting.

**Response:** Justification has now up revised in the background section. The last paragraph of the background has also been revised to convey the original thoughts of the authors and also satisfy the comments of the reviewer.

**Methods**

**Comment:** The figure under randomisation should be given a title and the source of it well provided. Reference should be made to the figure before introducing it.

**Response:** The manuscript has now been revised accordingly

**Providers**

**Comment:** Please, are you talking about health facilities or health providers?

**Response:** In this context, health facilities and health providers mean the same. This has been clarified in the manuscript to avoid any confusion.

**Healthcare financing in the study setting**

**Comment:**

Issues about healthcare financing should be moved to the study setting.

Out-of-pocket payments in the study setting

Issues about out-of-pocket payments should either be moved to the study setting or the background information.

**Response:** The section has been revised accordingly

**Results**

**Comment:** Authors should check the labelling of the tables due to inconsistencies identified during the review.

**Response:** Tables have now been appropriately labelled.

**Conclusions**

**Comment:** Please, state how relevant the findings are or what are the implications of the study findings.

**Response:** The conclusion has now been revised to include the implications of the study findings.

**Comment:** There are typographical issues that must be addressed. Therefore, proofreading the article will be very helpful.

Response: The entire manuscript has been proofread to address typographical issues
